# The Effects of Anthroposophic Medicine in Chronic Pain Conditions: A Systematic Review

**DOI:** 10.1089/jicm.2022.0723

**Published:** 2023-11-09

**Authors:** Markus Ploesser, David Martin

**Affiliations:** Fakultät für Gesundheit (Department für Humanmedizin), Lehrstuhl für Medizintheorie, Integrative und Anthroposophische Medizin, Herdecke, Germany.

**Keywords:** systematic review, anthroposophic medicine, chronic pain, effectiveness

## Abstract

**Background:**

The currently available evidence is unclear in regard to pain-related outcomes of patients with chronic pain conditions who undergo treatment with anthroposophic medicine (AM).

**Aim:**

To identify and synthesize the evidence in patients with chronic pain before and after AM therapy.

**Methods:**

The following databases and search interfaces were searched on October 21, 2021: Embase (via Embase.com), Medline (via PubMed), and the Cochrane Library. Additional references were identified via bibliographies of included studies. In at least one experimental arm that used anthroposophic therapy to treat chronic pain, AM treatments were required to be documented. Included studies reported on pain severity and physical and emotional functioning. Two authors independently assessed the studies for inclusion criteria, extracted the data, and conducted the quality evaluation of the included studies based on the critical appraisal tools provided by the Joanna Briggs Institute.

**Results:**

Seven studies (eight publications) were included in the review, of which were three randomized controlled trials (RCTs), two non-RCTs, and two pretest–post-test studies. A total number of 600 patients participated in the identified experimental studies, of whom all were adults. Three studies included patients with low back pain, one study each assessed patients with fibromyalgia, migraine, dysmenorrhea, and postpolio syndrome, respectively. The identified clinical studies reported considerable reductions in symptoms and effect sizes of pain outcomes after AM therapies being predominantly large, with no notable adverse effects.

**Conclusion:**

The findings of this systematic review of studies assessing AM therapies in patients with chronic pain problems revealed that there is a scarcity of evidence currently available, with unclear effects of AM treatments in reducing pain intensity and improving quality of life in the evaluated health conditions. Although most of the studies revealed a favorable benefit on one or more pain-related outcomes, the variability of the research did not allow for generalization across different studies, health conditions, and populations.

## Introduction

Chronic pain has been identified as one of the leading sources of disability worldwide.^1^ Prevalence rates differ widely, between 27% in the European Union and 43% in the United Kingdom, as well as 20% in the United States.^2–4^ It also has been regarded an important health issue, resulting in suffering of individuals, worsening of quality of life, and substantial economic and social cost, which approximate $560 billion annually in terms of direct medical expenditure, disability, and lost productivity in the United States.^5^

The administration of complex multidisciplinary interventions is necessary for the management of chronic pain, but the effectiveness of the widely used traditional techniques has been shown to be limited.^6^

Consequently, interest in using complementary and alternative therapies has been expanding to treat chronic pain and improve patients' physical, psychological, and social functioning.^6^

International Classification of Disorders (ICD)-11, the most recent version of the World Health Organization's ICD, comprises seven groups of the most prevalent clinically important diseases and defines chronic pain as persistent or recurrent pain lasting longer than 3 months:^7^ (1) chronic primary pain, (2) chronic cancer pain, (3) chronic post-traumatic and postsurgical pain, (4) chronic neuropathic pain, (5) chronic headache and orofacial pain, (6) chronic visceral pain, and (7) chronic musculoskeletal pain.

A person's physical function and psychological health can all be adversely affected by chronic pain.^8–11^ People who experience acute pain frequently develop chronic pain disorders, for example, up to 75% of patients with low back pain still suffer from this condition 12 months after the first episode, where the development of chronic pain is a result of a complex interplay between biological, psychological, and social factors.^12–14^ Anxiety, depression, pain catastrophizing, and fear of pain are all potential psychological risk factors for chronic pain.^15,16^

There is consensus that chronic pain is expected to get worse as the population ages.^17^ Furthermore, the impact of chronic pain on the lives of sufferers is profound, which can affect the ability to work, maintain relationships, and normal day-to-day functioning. An increased risk of developing comorbid health conditions in chronic pain sufferers, such as depression, obesity, and cardiovascular disease as well as cancer, has also been shown.^18,19^

The unmet needs regarding pain therapy are inadequate pain control and side effects associated with increased dosage of pain medications. Complementary and integrative health (CIH) treatments can assist people with chronic pain adjust to, accept, and manage their pain as well as deal with stresses in their daily lives that are related to their pain.^20^ For managing or reducing stress, enhancing coping, and promoting self-management, CIH practices include a variety of psychological (such as cognitive restructuring, problem-solving skills training, meditation), physical (such as progressive muscle relaxation, acupuncture), or combined psychological and physical (such as *t'ai chi*, yoga) approaches.^21^ U.S. data from 2012 indicate that ∼33% of adults and 12% of children (4–17 years old) have used CIH therapies, with the leading indication being chronic pain.^22,23^

Anthroposophic medicine (AM) is an integrative form of therapy (meaning that it integrates the whole of mainstream medicine with complementary therapies) initiated 100 years ago by Rudolf Steiner, PhD (1861–1924) and Ita Wegman, MD (1876–1943).^24^ AM considers itself an extension of conventional medicine, uses scientific methods, and also recognizes specific organismic, mental, and spiritual entities, and laws. It is salutogenically oriented and forms a unique concept of disease, therapy, and healing.^24^

The concept of AM sees the human organism not only created by physical and chemical laws, but by a total of four different forces: (1) the physical forces, (2) the growth forces that work in partnership with the physical forces, (3) a different category of forces (anima, soul) that combine with the physical and development forces to produce the sensory, motor, neurological, and circulatory systems' dualities of internal–external, (4) a fourth category of forces, known as Spirit, which supports human expression of the mind and the ability for reflective thought while interacting with the other three.^25^

A human system with three subsystems is created as a result of the interplay between these four systems of forces with active motor function and passive sensory perception: the nerve-sensory system, and the motor-metabolic system, which are polar to each other, and the intermediate rhythmic system.^26,27^

AM thus takes the spiritual, soul, vital, and physical aspects into account and integrates them into a resource-oriented medical understanding of health and illness, diagnostics, and therapy.^28^ This results in an individual patient approach, as these aspects are different for each patient and interact in different ways. It involves certain treatments and therapies including pharmaceutical, art, movement, and massage therapies, as well as specialized nursing approaches, and is presently used in more than 80 countries by doctors, therapists, and nurses.^28^ The therapeutic objectives include promoting self-healing, developing autonomic regulation of the organism, and promoting psycho-emotional, spiritual, and other forms of self-regulation.^27^ To achieve this aim, all dimensions of the sick patient are taken into account, including the physical, emotional, mental, spiritual, and social aspects, which increase the chances of improvement.^27,29^

The therapeutic methods provided by AM include a variety of customized mineral, plant, and animal remedies, several art therapies (such as painting therapy, sculpting, music therapy, speech therapy), rhythmical massage, eurythmy therapy (body movements with associated contemplative features, performed in a group setting), external remedies (compresses, baths, ointments), counseling and medical consultations, and anthroposophically focused care. These techniques are meant to energize and fortify the patient's inherent healing abilities and are used by doctors, nurses, and therapists. AM doctors get training in both AM and standard medical practice.^30–32^

Previous research in patients with chronic low back pain showed comparative improvements in pain symptoms compared with conventional therapy.^33^ Another study investigating AM in patients with chronic disease (including mental health and musculoskeletal impairments) found a sustainable improvement over a 2-year follow-up time.^34^ To date, no systematic reviews investigating the effects of AM in patients with chronic pain have been identified.

The objective of the present systematic review is to assess the impact and safety of anthroposophic interventions in the treatment of chronic pain conditions.

## Methods

This systematic review is reported according to the Preferred Reporting Items for Systematic Reviews and Meta-Analyses.^35^

The following criteria according to PICOS (participants, intervention, comparators, outcomes, study design) apply.

### Participants

Adult patients (≥18 years old) with chronic pain of >3 months were included.

### Interventions

Studies in English and German were included, and at least one study arm used AM treatment to treat chronic pain.

### Comparators

The following comparators were included: standard treatment, placebo, or waiting list control.

### Outcome measures

Pain severity and physical and emotional functioning are specified as core domains in chronic pain treatment trials, according to the Initiative on Methods, Measurement, and Pain Assessment in Clinical Trials (IMMPACT).^36,37^ The primary outcome was pain severity, measured with a validated self-rating instrument, for example, the Visual Analog Scale (VAS, 0–10/0–100) or the Numeric Rating Scale (NRS, 0–10).^38^ Secondary outcomes included health-related quality of life, pain-related disability, mood (e.g., depression, anxiety, pain catastrophizing, and pain-related fear), and adverse events.

### Study design

Randomized controlled trials (RCTs) and quasi-experimental studies (nonrandomized studies and pre–post-test studies) meeting the following criteria were included: assess the impact of AM to treat chronic pain, describe the intervention in great detail, published in a peer-reviewed Journal.

### Electronic searches

English and German articles were searched on October 21, 2021 in MEDLINE (via Pubmed.com), Embase (via Embase.com), and the Cochrane Central Register of Controlled Trials (CENTRAL) (via the Cochrane Library). To minimize the risk of selection bias, English abstracts of articles in other languages were screened to determine suitability for translation and inclusion into the review. Articles with abstracts in any other language than English or German were excluded. Reviews and retrieved publications' reference lists were checked for any further research to incorporate. To obtain additional study information, authors of publications were contacted when necessary. So-called “gray literature” (i.e., Doctoral and Master dissertations) was searched through Google Scholar. Search terms included various keywords for chronic pain syndromes, AM, and study designs. Medical subject headings (MeSH) were used wherever possible. The following shows the Embase search strategy: (“chronic pain”/exp OR “chronic pain” OR “complex regional pain syndrome”/exp OR “complex regional pain syndrome” OR “chronic low back pain”/exp OR “chronic low back pain” OR “fibromyalgia”/exp OR “fibromyalgia” OR “facetogenic pain” OR “chronic osteoarthritis” OR “failed back surgery syndrome”/exp OR “failed back surgery syndrome” OR “central pain syndrome”/exp OR “central pain syndrome” OR “neuralgia”/exp OR “neuralgia” OR “neuropathy”/exp OR “neuropathy” OR “post stroke pain”/exp OR “post stroke pain” OR “chronic cancer pain” OR “chronic migraine”/exp OR “chronic migraine”) AND (anthropos* OR weleda OR wala OR “curativ* and eurythm*” OR “rhyhmic* and massage” OR “rhythmic and massage” OR “pressel and massage” OR eurythm* OR infludo OR nausyn OR “cardiodoron”/exp OR cardiodoron OR combudoron OR hepatodoron OR choleodoron OR digestodoron OR dermatodoron OR pneumodoron OR pneumadoron OR erysidoron OR kephalodoron OR cephalodoron OR biodoron OR “ferrum and quar*” OR menodoron OR pertudoron OR echinadoron OR biodor OR onopordon OR bidor OR venadoron OR “plantago and bronchial*” OR “bolus and eucalypt* and comp*” OR chirophoneti* OR “bothmer* and gymnasti*” OR “mistletoe or mistletoe*” OR “viscum or viscum*” OR “iscador or iscador*” OR “iscar or iscar*” OR “helixor or helixor*” OR “iscucin or iscucin*” OR “isorel or isorel* or visorel or visorel*” OR abnoba* OR “waldorf or waldorf*” OR “rudolf and steiner”) AND (study* OR studie* OR “trial”/exp OR trial OR trial* OR evaluat* OR random* OR investig* OR cohort* OR kohort* OR outcome* OR “review”/exp OR review OR review* OR ubersicht OR uebersicht OR übersicht OR uberblick OR ueberblick OR überblick OR metaanalys* OR “meta analys*” OR “meta and analys*”).

### Data collection and analysis

Eligibility of studies for inclusion were determined by two independent reviewers, by reviewing the study's abstracts. Disagreements between reviewers were resolved by discussion. Data extraction was performed by two independent review authors into predefined data extraction tables. Discrepancies in data extraction were resolved discussion.

Extracted data included study information (title, authors, year, and country), study population (participant sociodemographic and clinical characteristics, initial and final sample size), information about the interventions and controls (type, dosage, treatment session durations, treatment session frequencies), outcomes (measures, scores), and dropouts.

### Quality assessment

The critical appraisal of the included studies was carried out by means of the checklist for quasi-experimental studies and RCTs, provided by the Joanna Briggs Institute.^39^ These checklists consist of several steps to answer specific questions about the study methodology that aid to establishing the methodological rigor as well as the potential risk of bias of the study in question. Results of the appraisal of the included studies are summarized in a corresponding table.

The protocol for this systematic review has not been published, and the review is not registered with a systematic review database.

## Results

### Study searches and selection

**FIG. 1.**
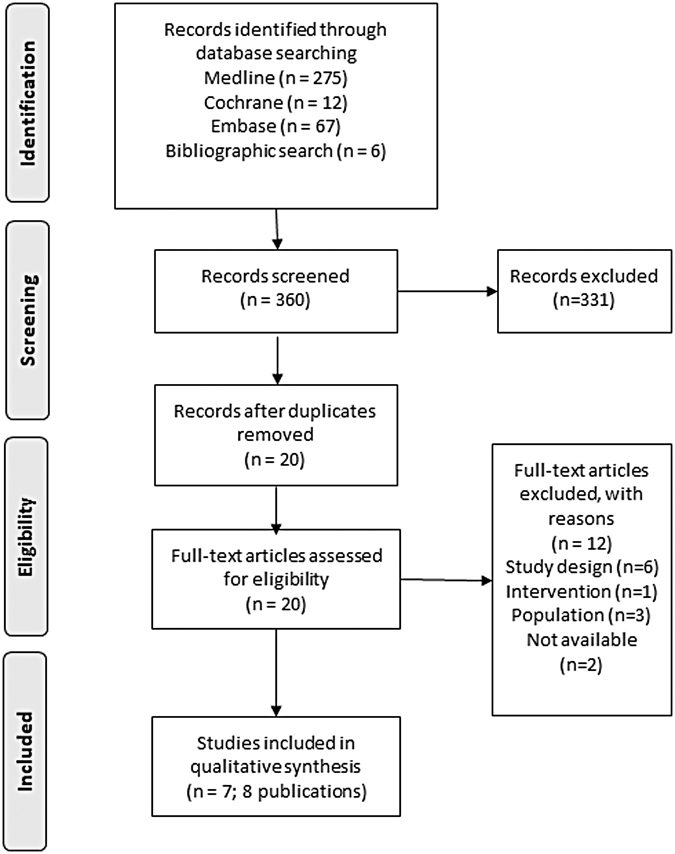
PRISMA flow diagram.

The systematic database search yielded a total of 360 records. After removal of duplicates and excluding studies as per eligibility criteria, 20 full-text articles were assessed for inclusion in this review. After exclusion of 12 articles, 7 final studies (8 publications) were included in this review (Fig. 1).

### Study characteristics

Of the seven included studies, three were RCTs, two were non-RCTs, and two studies were experimental studies with pre- and post-test design. One of the five studies was available in abstract form only.^40^ Only four studies were identified that were conducted in the past 10 years.^40,41–43^ One study (Hamre et al.^33,44^) was split in two publications, of which the one with the later publication year reported on a 24-month follow-up.

The number of participants ranged from *N* = 30 to *N* = 298. A total number of 600 patients participated in the identified experimental studies, of whom all were adults. Three studies included patients with low back pain,^33,40,41,44^ one study each assessed patients with fibromyalgia,^45^ migraine,^46^ dysmenorrhea,^43^ and postpolio syndrome,^42^ respectively.

The duration of the intervention was in the range of 2–3 months for all studies, and two studies assessed long-term follow-up up to 24 months.^33,44,46^ One study did not report on the type of practitioner carrying out the intervention.^40^

Six of the seven studies reported on outcome parameters and measures, with pain ratings and quality of life most commonly assessed through self-report measures.

The reporting on adverse events occurred only in four studies.^33,41,42,44,46^ The RCT by Michalsen et al. reported mostly mild and self-limited exacerbating back pain, of which 3 cases occurred in the AM eurythmy therapy group, compared with 7 cases in the yoga group and 25 cases in the physiotherapy group.^41^ In the study by Ghelman et al., no adverse events were observed.^42^ In the study by Hamre et al., 3% (*n* = 1) of those taking anthroposophic drugs and 9% (*n* = 3) of patients taking nonanthroposophic drugs reported adverse events.^46^ These included skin blisters from estradiol/norethisterone pills in individuals receiving nonanthroposophic drugs, nightmares from amitriptyline, and psychological symptoms from homoeopathic arsenicum album.^46^ The one patient taking anthroposophic medication (rosemary ointment) had to stop the application due to skin blisters. No serious adverse events occurred from medication use, and no side effects from nondrug treatments occurred.^46^

In the study by Hamre et al., adverse reactions occurred in 15% (5/34) of patients treated with AM, and 11% of patients in the conventional therapy group.^33,44^ No serious adverse events occurred.

### Therapeutic effects

First, we analyzed the three RCTs. The first study was a 3-arm RCTs on the clinical effects of three 8-week programs in 274 patients with chronic low back pain, including yoga, eurythmy therapy, and physiotherapy.^41^ Patients were included if they were aged between 18 and 70 years, had nonspecific back pain for at least 3 months including a medical specialist's written diagnostic confirmation, and if their back pain intensity was at least 40 mm on the 100 mm VAS on at least 4 of 7 weekdays. The primary outcome was physical disability assessed by the Robert Morris Disability Questionnaire (RMDQ) from baseline to week 8.^41^ Secondary outcome variables were pain intensity assessed by the VAS, health-related quality of life (SF-12) and life satisfaction. The outcome assessment was conducted at baseline, after the intervention at 8 weeks and at a 16-week follow-up. Results of this study show no significant differences between the three groups for the primary and all secondary outcomes, where in all groups, RMDQ decreased comparably at 8 weeks, but did not reach clinical meaningfulness.^41^

The study by Ghelman et al. was a 3-month, four-arm, randomized, double-blind, placebo-controlled, phase 2 prospective clinical trial referring to patients with chronic pain due to postpolio syndrome.^42^ As given in Tables 1 and 2, the authors reported significant improvement of pain symptom scores with the anthroposophic treatments as add-on to both, placebo (*d* = 1.315), or experimental transdermal gel (ETG) use (*d* = 2.035) (In the original publication we have found that the confidence interval is given as a single value rather than a range. We have tried to contact the author of the article but did not get a reply). ETG use, specifically when used in conjunction with the anthroposophic therapies, facilitated an improvement in quality of life and resilience.^42^

**Table 1.** Study Design and Interventions

Study	Study design, patients	Groups (*n*)	Intervention type	Duration of intervention	Who carried out the intervention
Michalsen et al.^41^ (Germany)	RCT (*N* = 298) chronic low back pain	Group 1: yoga (*n* = 100) Group 2: eurythmy (*n* = 92) Group 3: physiotherapy (*n* = 82)	Group 1: specific yoga techniques Group 2: specific eurythmy exercises Group 3: specific physiotherapy exercises	8 Weeks for all groups	Group 1: certified yoga instructor Group 2: certified eurythmist Group 3: physiotherapist
Ghelman et al.^42^ (Brazil)	RCT (*N* = 40) Postpolio syndrome	Group 1: ETG (*n* = 10)^a^ Group 2: PTG (*n* = 10)^b^ Group 3: ETG plus anthroposophic therapy^c^ (*n* = 10) Group 4: PTG plus anthroposophic therapy^c^ (*n* = 10)	Group 1: 1 g of ETG nightly in the painful regions Group 2: 1 g of PTG nightly in the painful regions Group 3: 1 g of ETG nightly in the painful regions plus one weekly session of AAT (1 h) + PMRN/AET (1 h)^*^ Group 4: 1 g of PTG nightly in the painful regions plus one weekly session of AAT (1 h) + AET/PMRN (1 h)^*^ Patients continued to receive their usual medical care, such as analgesics	12 Weeks for all groups	PMRN: licensed speech therapist AET: registered nurse NR for the other therapies
Vagedes et al.^43^ (Germany)	RCT (*N* = 60) dysmenorrhea	Group 1: RMT (*n* = 23) Group 2: biofeedback (*n* = 20) Group 3: usual care (*n* = 17)	Group 1: RMT including effleurage with light, rhythmical engagement into the tissues, kneading with circular movements of varying speed, depth and intensity Group 2: biofeedback using the HRV Qiu (BioSign GmbH, Ottenhofen, Germany) Biofeedback device Group 3: usual care mainly consisting of analgesic medication, physical exercise and application of warmth	12 Weeks for all groups	Group 1: anthroposophic RMT therapist Group 2: at home by patient Group 3: patient
Baars and Ellis^45^ (The Netherlands)	Pretest–post–test (*N* = 41) Fibromyalgia	N/A	Weekly dose of 10 mL Hepar Magnesium D10, intravenously	10 Weeks	Anthroposophic general practitioner
Hamre et al.^46^ (Germany)	Pretest–post-test (*N* = 45) Migraine	N/A	Medications (67% of patients), eurythmy therapy (38%), art therapy (18%), and RMT (13%)	Median 105 days	Anthroposophic general practitioner
Enrico et al.^40^ (Italy)	Non-RCT (*N* = 30) Vertebrolumbar pain (back pain)	Group 1: after initial therapy, no further treatment (*n* = 15) Group 2: after initial therapy further treatment (*n* = 15)	Arnica planta tota Rh D3 fl 1 a day subcutaneously at points of maximum pain Group 1: subsequently, 15 patients did not receive any pain treatment Group 2: 15 patients continued treatment with the same anthroposophic remedy	Initial treatment for both groups: 6 days Group 2: twice-weekly dosing for 3 months	NR
Hamre et al.^33,44^ (Germany)	Nonrandomized controlled study (*N* = 86) Low back pain	Group 1: Conventional therapy (*n* = 48) Group 2: anthroposophic therapy (*n* = 38)	Group 1: Conventional therapy Group 2: anthroposophic therapy (eurythmy, rhythmical massage or art therapy; counseling, anthroposophic medication)	Median therapy duration was 88 (range 59–123) days, median number of therapy sessions was 12 (range 10–12)	Group 1: general physicians Group 2: anthroposophic physician

^a^ The active ingredients of the 10% ETG were Rhus toxicodendrum D4 (1.66%), Arnica montana D3 (1.66%), Apis mellifica/Atropa belladonna D3 (0.83%/0.83%), Mandragora officinarum D3 (1.66%), Aconitum napellus D4 (1.66%), and Hypericum perforatum D3 (1.66%)

^b^ The PTG vials contained only the inert excipient soy lecithin.

^c^ Group 3 and Group 4: All the patients started the interventions together in a single group by receiving AAT, after which half of the group received PMRN and the other half received the ^*^AET, and then, the therapies were alternated between the groups.

^*^ AAT: The technique used was watercolor painting, according to the guidelines of the Brazilian, Dutch, and Swiss Association of Art Therapists.

^*^ PMRN: The therapeutic activity was conducted in two stages, including body and oral exercises of reflex-vegetative functions. The first stage included a sequence of eight body exercises, and the second stage included a sequence of 14 body exercises.

^*^ AET: This program involved the performance of a sequence of three techniques, with a total duration of 15–20 min each; a restful state was stimulated after the program through a sequence of three procedures: warm water footbath, rhythmic massage, compresses.

AAT, anthroposophic artistic therapy; AET, anthroposophic external therapy; ETG, experimental transdermal gel; HRV, heart rate variability; N/A, not applicable; NR, not reported; PMRN, Padovan method of neurofunctional reorganization therapy; PTG, placebo transdermal gel; RCT, randomized controlled trial, RMT, rhythmical massage therapy according to I. Wegman.

**Table 2.** Outcomes and Results

Study	Study outcomes and measures	Measurement timepoints	Lost to FU (*n*)	Main results
Michalsen et al.^41^ (Germany)	Disability (RMDQ): Pain (VAS): QoL (SF-12): life satisfaction (BMLSS)	T0: Baseline T1: 8 weeks T2: 16 weeks	Group 1: *n* = 64 Group 2: *n* = 59 Group 3: *n* = 56	No superiority of yoga or eurythmy therapy over physiotherapeutic exercises could be demonstrated. Patients' physical disability (RMDQ) showed no significant between-group differences. The secondary outcomes showed no significant between-group differences, but comparable improvements of symptoms, function, and quality of life in all three intervention groups
Ghelman et al.^42^ (Brazil)	Pain (VAS, McGill Questionnaire, transcutaneous thermography); QoL (WHOQOL-BREF); Resilience (QSCA)	T0: Baseline T1: 12 weeks	Group 1: *n* = 1 Group 2: *n* = 2 Group 3: *n* = 2 Group 4: *n* = 1	In groups 3 and 4, pain reduction was statistically significant in both the placebo group (*p* = 0.02, *d* = 1.315) and in the ETG (*p* = 0.005, *d* = 2.035). In the groups that used ETG, especially when associated with the therapies, there was an improvement in both the quality of life and the degree of resilience
Vagedes et al.^43^ (Germany)	Pain (NRS), Use of analgesics (diary), HRV parameters (24-h ECG); QoL (SF-12)	T0: Baseline T1: 12 weeks	Group 1: *n* = 2 Group 2: *n* = 6 Group 3: *n* = 4	Significant difference was only seen between rhythmical massage and control group in pain outcome (mean difference: −1.61; 95% CI: −2.77 to −0.44; *p* = 0.004), but not between biofeedback and control group. No differences seen in use of analgesics, HRV, and QoL between groups
Baars and Ellis^45^ (The Netherlands)	Symptoms (FIQ, Dutch translation)	T0: Baseline T1: 5 weeks T2: 10 weeks	*n* = 8 at 10 weeks	Total FIQ score improved by at least 20% in 41.5% and 50% of patients after 5 and 10 weeks, respectively
Hamre et al.^46^ (Germany)	Migraine severity (NRS; physician and patient rated); symptoms (NRS), QoL (SF-36)	T0: Baseline T1: 3 months T2: 6 months T3: 12 months T4: 18 months T5: 24 months	*n* = 20 at 24 months	From baseline to 6-month follow-up, physician-rated average migraine severity improved by 3.14 points (95% CI: 2.40–3.87, *p* < 0.001); patient-rated average migraine severity improved by 2.82 points (2.05–3.64, *p* < 0.001); and symptom score improved by 2.32 points (1.68–2.95, *p* < 0.001). Three SF-36 scales (Social Functioning, Bodily Pain, Vitality), the SF-36 Physical Component summary measure, and the SF-36 Health Change item improved significantly. All improvements were maintained at last follow-up after 24 months
Enrico et al.^40^ (Italy)	NR	T0: Baseline T1: 1 month T2: 2 months T3: 3 months	NR	Arnica planta tota Rh D3 vials resulted in a significant improvement in both groups during the acute phase. The analysis of follow-up after 1, 2, and 3 months showed that the group treated with Arnica planta tota Rh D3 had a maintained clinical benefit, which was not the case for the control group
Hamre et al.^33,44^ (Germany)	Functional disability (HFAQ); pain (LBPRS); symptom score (NRS); QoL: SF-36	T0: Baseline T1: 6 months T2: 12 months Group 2 only: T3: 24 months	Group 1: *n* = 20 Group 2: *n* = 4	Significant improvements in both groups of HFAQ, LBPRS, symptom score, SF-36 physical component summary, and three SF-36 scales (physical function, pain, vitality), and improvements in group 2 patients of three further SF-36 scales (role physical, general health, mental health) at 12 months At 24 months, patients with chronic LBP receiving anthroposophic treatment had sustained improvements of symptoms, back function, and quality of life

BMLSS, Brief Multidimensional Life Satisfaction Scale; CI, confidence interval; ECG, electrocardiogram; FIQ, Fibromyalgia Impact Questionnaire; FU, follow-up; HFAQ, Hanover Functional Ability Questionnaire; HRV, Heart Rate Variability; LBPRS, Low Back Pain Rating Scale Pain Score; NRS, Numeric Rating Scale; RMDQ, Roland Morris Disability Questionnaire; SF; Short Form; T, Timepoint; QoL, quality of life; QSCA, Antonovsky's sense of coherence questionnaire; VAS, visual analog scale; WHOQOL-BREF, The World Health Organization Quality of Life Questionnaire.

All other published clinical outcome details of this study are summarized in Tables 1 and 2. The authors of this study concluded that the multimodal anthroposophic treatment was both safe and efficacious as an analgesic in the groups that received anthroposophic therapies as a stand-alone intervention but showed these properties much sooner when combined with the ETG than when used alone. There was an improvement in both the quality of life and the degree of resilience in the groups that used ETG, especially when it was used in conjunction with the anthroposophic treatments. Hence, the anthroposophic treatment's multimodal usage was found to be effective for pain reduction.^42^

The third study with a controlled design investigated 60 patients with dysmenorrhea investigating rhythmic massage compared with biofeedback and usual care.^43^ The authors measured pain, drug use, heart rate variability, and quality of life. Treatment effects were only seen between the rhythmical massage according to Ita Wegman^47^ and control group in the pain outcome.^43^ All other comparisons and outcomes showed nonsignificant differences. The overall results of the study reflect an effective rhythmic massage modality to improve pain intensity of dysmenorrhea after 12 weeks (Table 2).^43^

Two studies had a control group, but were nonrandomized.^33,40,44^ Hamre et al. engaged 86 patients with persistent low back pain in a prospective nonrandomized comparative study, comparing anthroposophic therapy in the form of eurythmy, rhythmical massage, or art therapy, counseling, or AM with conventional therapy (Table 1).^33,44^ Both groups showed substantial improvement in symptom ratings and physical health, with the AM group showing more dramatic gains in mental health, general health, and energy at the first follow-up at 12 months. At 24 months, patients with chronic LBP receiving anthroposophic treatment had sustained improvements of symptoms, back function, and quality of life (Table 2).^33,44^ The study's sample size was small, with 38 patients in the anthroposophic treatment group and 48 in the traditionally treated group.^33,44^

The other study was only available as an abstract,^40^ investigating Arnica planta tota Rh D3s.c. initially in 30 patients with back pain, of whom 15 patients continued over a period of 3 months, whereas the other 15 patients did not continue. Results indicated that in the initial treatment period of 6 days for all patients, significant improvements were seen in both groups. While on the continuing treatment group these clinical benefits were maintained, whereas in the group that did not continue taking Arnica planta, symptoms returned (Table 2).^40^

Two exploratory pre–post studies without a control group were also analyzed.^45,46^ The study by Baars and Ellis investigated Hepar Magnesium D10 in a sample of 41 patients with fibromyalgia over 10 weeks.^45^ The authors reported a total Fibromyalgia Impact Questionnaire (FIQ) score improvement by at least 20% in 41.5% and 50% of patients after 5 and 10 weeks, respectively (Table 2). After 10 weeks of treatment, Cohen's delta effect size was 0.68 (medium effect, no confidence interval reported by the authors).^45^

Hamre et al. conducted a real-world pre–post study in 45 migraine patients, investigating anthroposophic medications, eurythmy, art, and rhythmical massage in 67%, 38%, 18%, and 13% of patients, respectively.^46^ At the 6-month follow-up, certain quality-of-life scale items as well as the physician and patient-rated migraine intensity both showed significant improvements, which were sustained at the 24-month follow-up (Table 2).^46^

The study characteristics and results of the included trials are summarized in Tables 1 and 2.

### Critical appraisal of included studies

For the three RCTs, most of the critical appraisal questions could be answered with a “Yes,” indicating that the quality of these trials was satisfactory. For the questions that were answered as “Unclear,”^42^ we have contacted the authors to clarify, but did not receive a response (Table 3).

**Table 3.** Critical Appraisal of Randomized Controlled Trials (According to Joanna Briggs Institute)

Study	1	2	3	4	5	6	7	8	9	10	11	12	13
Michalsen et al.^41^ (Germany)	Y	Y	Y	N/A	N/A	Y	Y	Y	Y	Y	Y	Y	Y
Ghelman et al.^42^ (Brazil)	Y	Y	U	N/A	N/A	U	Y	Y	Y	Y	Y	Y	Y
Vagedes et al.^43^ (Germany)	Y	Y	Y	N/A	N/A	N	Y	Y	Y	Y	Y	Y	Y

1. Was true randomization used for assignment of participants to treatment groups?; 2. Was allocation to treatment groups concealed?; 3. Were treatment groups similar at the baseline?; 4. Were participants blind to treatment assignment?; 5. Were those delivering treatment blind to treatment assignment?; 6. Were outcomes assessors blind to treatment assignment?; 7. Were treatment groups treated identically other than the intervention of interest?; 8. Was follow-up complete and if not, were differences between groups in terms of their follow-up adequately described and analyzed?; 9. Were participants analyzed in the groups to which they were randomized?; 10. Were outcomes measured in the same way for treatment groups?; 11. Were outcomes measured in a reliable way?; 12. Was appropriate statistical analysis used?; 13. Was the trial design appropriate, and any deviations from the standard RCT design (individual randomization, parallel groups) accounted for in the conduct and analysis of the trial? (Authors were contacted if unclear from publication.)

Y, yes; N, no; N/A, not applicable; U, unclear; N/A, not applicable.

For the studies of pre- and post-test design,^45,46^ most of the questions were answered with “Yes,” indicating that by design these studies were largely methodologically sound. *Per definitionem*, a study of pre- and post-test design does not have a control group, hence both studies answered question 4 with “No” (Table 4).

**Table 4.** Critical Appraisal of Quasi-Experimental Studies (According to Joanna Briggs Institute)

Study	1	2	3	4	5	6	7	8	9
Baars and Ellis^45^ (The Netherlands)	Y	Y	Y	N/A	Y	Y	Y	Y	Y
Hamre et al.^46^ (Germany)	Y	Y	Y	N/A	Y	Y	Y	Y	Y
Enrico et al.^40^ (Abstract only) (Italy)	Y	Y	Y	Y	Y	U	Y	U	U
Hamre et al.^33,44^ (Germany)	Y	Y	Y	Y	Y	Y	Y	Y	Y

1. Is it clear in the study what is the “cause” and what is the “effect” (i.e., there is no confusion about which variable comes first)?; 2. Were the participants included in any comparisons similar?; 3. Were the participants included in any comparisons receiving similar treatment/care, other than the exposure or intervention of interest?; 4. Was there a control group?; 5. Were there multiple measurements of the outcome both pre and post the intervention/exposure?; 6. Was follow-up complete and if not, were differences between groups in terms of their follow-up adequately described and analyzed?; 7. Were the outcomes of participants included in any comparisons measured in the same way?; 8. Were outcomes measured in a reliable way?; 9. Was appropriate statistical analysis used?

Y, yes; N, no; U, unclear; N/A, not applicable.

For the nonrandomized studies,^40,33,44^ some questions were rated as unclear since the study by Enrico et al. was only supplied as an abstract, which lacked information of methodology. Otherwise, the study by Hamre et al. could be rated with a “Yes” for all answers (Table 4).^33,44^

## Discussion

The aim of this systematic review was to assess the impact and safety of anthroposophic interventions in the treatment of chronic pain conditions. This is the first systematic review of practice-based (or real-world) trials of AM therapies for chronic pain syndromes of patients that have been administered in outpatient or specialty anthroposophic clinics. Results indicate a scarcity of studies in general, with only four studies identified that were conducted in the past 10 years. Reasons for this could be that research into nonpharmacologic treatments like anthroposophic therapy does not obtain adequate funding, and hence, well-designed controlled trials are rare.

Within the few studies that were found, the indications, research designs, outcome measures, and the use of supplementary therapies displayed significant heterogeneity. Nonetheless, several of the publications included here were of acceptable quality and presented data in an appropriate manner. The retrieved abstract, on the contrary,^40^ had methodological reporting shortcomings by nature, therefore the reported results must be interpreted with caution. In addition, the distinct background of the therapists might be a possible risk of bias that may be difficult to rule out; nonetheless, most publications on unique complementary and integrative treatments will have this bias.

In general, the identified clinical studies report considerable reductions in symptoms and effect sizes of pain outcomes after AM therapies being reported in five studies and being predominantly large (<0.8), which may be attributed to the benefits of the different anthroposophic therapy methods, with no notable adverse effects. While statistical significance is commonly reported in most trials, the reporting of effect sizes in the included studies is encouraging since it provides a clearer indicator of the degree of change and has recently been proposed as a standard component of results reporting.^48,49^

However, a large effect size in the majority of included studies can also be an indication of publication bias, which usually is assessed as part of a meta-analysis but this was not done in this systematic review. Since we also have searched for gray literature as part of this systematic review, we have incorporated at least this particular suitable measure to account for publication bias.^50^ Another factor contributing to moderate to large effect sizes in individual studies may be underpowered studies, when in fact, the true effect is small or moderate and its magnitude is being overestimated.^51^

Of the seven studies included, four studies (two RCTs and two nonrandomized studies) assessed comparative effectiveness and only one RCT investigated efficacy, whereas the other two studies were single-arm studies that are unable to specify effectiveness or efficacy of an intervention. While both efficacy and effectiveness research is crucial for evaluating therapeutic interventions, their objectives and research methodologies are different. Efficacy studies investigate an intervention's advantages and disadvantages under rigorous guidelines. Although this has many methodological benefits and produces high internal validity, it necessitates significant deviation from clinical practice, including restrictions on the patient sample, control of the provider skill set, and limitations on provider actions, as well as elimination of multimodal treatments.^52^ The best design for evaluating efficacy is a placebo-controlled RCT, which reduces bias through a number of processes, including double blinding and uniformity of the intervention.

RCTs often resolve problems with patient acceptability and adherence, physician endorsement, and access (the intervention is offered for free). On the contrary, effectiveness studies, commonly referred to as pragmatic studies, analyze therapies in conditions that are more analogous to real-world practice, including dispersion in typical clinical settings, more diversified patient groups, and less stringent treatment protocols.^52^ RCT designs may also be used in effectiveness studies, although more frequently than not, the intervention is compared with standard care rather than a placebo. There are only a few restrictions on the provider's ability to alter the dosage, the dosing schedule, or co-therapy, allowing for customized treatment for each patient. Effectiveness studies have more external validity than efficacy trials, despite significant internal validity sacrifices. The absence of an observed impact in effectiveness studies might be caused by a number of variables, such as poor execution, ineffective treatment, lack of physician approval, or lack of patient acceptance and adherence.^52^

According to the above-mentioned definition, most of the controlled studies in this systematic review examined effectiveness (arguably Ghelman et al.^42^ could be classified as an efficacy trial), and thus the real-world effects of AM.

Regarding the selection of a placebo/sham control for clinical research in CIH, there are a number of distinct methodological difficulties. A sham control needs to capture the “nonspecific” aspects of the therapy while leaving out the “specific” aspects related to the current study topic to be a useful comparison. But creating such a false intervention is not always simple. Other crucial aspects of making the right design decisions include accounting for social interaction between practitioners and subjects, addressing ethical concerns, having accurate measurements of treatment integrity and intensity, and choosing practitioners with sufficient experience when conducting the sham process.^53^

Because several studies used a mixture of anthroposophic treatments,^33,42,44^ it may be difficult to assess the individual contributions of the single anthroposophic treatment modalities that make up the respective complex intervention. In reality, the investigations examined the effects of seeking out an anthroposophic therapist who then provides their real-world treatment, which includes among the actual anthroposophic treatment component also the nonspecific treatment effects, such as placebo effects, practitioner–patient relationships, the practice environment, and patient expectations, which are vastly different for each patient.

Patients with low back pain accounted for a total of 414 patients and were thus the most researched patient population.^33,40,41,44^ Although in the study by Hamre et al.^33,44^ the inclusion criteria was to have low back pain for at least 6 weeks and in the study by Enrico et al.^40^ there was no mention how long the participants had to have the back pain before inclusion into the study, one could argue that recovery as part of the natural history of disease may be possible within the 12 weeks of treatment period, but unlikely as a control group in both studies was included, although without randomization. The same goes true for the RCTs.^41–43^

For the two pre–post-test studies, the absence of a control group limits proof of effectiveness versus natural course or other treatments.^45,46^ In addition, the initial patient numbers per study was <100 in most cases, and some of the studies suffered significant dropout rates that contribute to further concern of bias.

According to research conducted in primary health care settings, both patients and practitioners are aware that AM therapies cover gaps in treatment effectiveness for patients suffering from severe chronic conditions.^54^ More practice-based approaches could be used for evaluating AM effectiveness in the real-world clinical context as addition to using RCTs with prespecified patient demographics and study methods.^55^

Because chronic pain affects many people throughout the world, more practice-based research is needed to develop an effective and individualized treatment regimen (including dose and timing of therapies) for this patient population. While several pain outcome measures were included in the identified studies, their inclusion varied among trials, making it difficult to compare pain changes across trials. Future research should utilize an 11-point NRS for pain severity to make it simpler to compare data between studies. Given that it is a validated measure, it can be used to assess pain in situations when time is of the essence or if a speedy evaluation is required.^56–59^

One of the drawbacks of the included studies was a lack of information about the participants' socioeconomic status or ethnicity, which made it impossible to determine whether the findings were generalizable to a wider population. Including such demographic characteristics in future research will allow for a more in-depth evaluation of this variable's significance as a possible predictor of response.

Larger sample sizes and more clinical study sites should be included in future studies where possible to better reflect the general patient and clinic population, and to ensure that the study findings are generalizable to clinical patients and clinicians.^60^ In addition, the bulk of the included studies did not employ multivariate analysis to discover potential predictive elements in their findings. It is understandable that multivariate analysis is not appropriate for research using lower sample sizes due to the statistical limitations of the method. For more meaningful results in future evaluations, it is recommended that multivariate analysis be performed if the sample size of the study allows it to better understand how various baseline characteristics (e.g., intensity of pain, duration of pain, demographics) are associated with responsiveness on pain outcomes following different AM interventions.^61^

There were certain limitations to the current systematic review. For example, because of the heterogeneity in research design and the weak reporting in the publications, it was difficult to make comparisons between studies and draw conclusions. Only English and German language articles were included, which may have led to the exclusion of relevant articles in other languages.

In terms of future AM research, the following recommendations are made: A more consistent and comprehensive reporting system is required, for example, based on the recommendations for research reporting made by the Enhancing the Quality and Transparency of Health Research (EQUATOR) guidelines according to study design, which may also improve the collective understanding of how to most effectively apply AM treatments in clinical settings.^62^

The outcomes were assessed within 2–24 months of treatment initiation, whereby examining long-term outcomes of >6 months is recommendable for future studies.

To provide a choice of interventions that have been proven to be successful for patients' clinical conditions in the past, future AM practice-based efforts should include an increased number of study centers and a more diversified collection of patient-reported pain outcomes. This will allow for the formulation of optimal AM pain treatments tailored to the requirements and features of specific patients.

## Conclusions

In conclusion, the findings of this systematic review of studies assessing AM therapies in patients with chronic pain problems revealed that there is a scarcity of evidence currently available, with unclear effects of AM treatments in reducing pain intensity and improving quality of life in the evaluated health conditions. Although some of the studies revealed a favorable benefit on one or more pain-related outcomes, the variability of the research did not allow for generalization across different studies, health conditions, and populations. This study has revealed that more practice-based research on AM treatments for this patient population is essential to inform clinical practice in the real-world setting.
